# Engineered exosomes: desirable target-tracking characteristics for cerebrovascular and neurodegenerative disease therapies

**DOI:** 10.7150/thno.62330

**Published:** 2021-08-18

**Authors:** Meng Xu, Tao Feng, Bowen Liu, Fen Qiu, Youhua Xu, Yonghua Zhao, Ying Zheng

**Affiliations:** 1 State Key Laboratory of Quality Research in Chinese Medicine, Institute of Chinese Medical Sciences, University of Macau, Taipa, Macau, China.; 2 Faculty of Chinese Medicine, State Key Laboratory of Quality Research in Chinese Medicine, Macau University of Science and Technology, Taipa, Macao, China.

**Keywords:** engineered exosomes, brain targeting, tracking, administration routes, cerebrovascular disease, neurological disorders

## Abstract

As extracellular vesicles secreted by cells, exosomes are intercellular signalosomes for cell communication and pharmacological effectors. Because of their special properties, including low toxicity and immunogenicity, biodegradability, ability to encapsulate endogenous biologically active molecules and cross the blood-brain barrier (BBB), exosomes have great therapeutic potential in cerebrovascular and neurodegenerative diseases. However, the poor targeting ability of natural exosomes greatly reduces the therapeutic effect. Using engineering technology, exosomes can obtain active targeting ability to accumulate in specific cell types and tissues by attaching targeting units to the membrane surface or loading them into cavities. In this review, we outline the improved targeting functions of bioengineered exosomes, tracing and imaging techniques, administration methods, internalization in the BBB, and therapeutic effects of exosomes in cerebrovascular and neurodegenerative diseases and further evaluate the clinical opportunities and challenges in this research field.

## Introduction

Central nervous system (CNS) disorders are complex and life-threatening diseases with lack of ideal clinical treatment techniques, which can lead to disability and death with high probability. Among them, stroke is the most common cause of permanent disability in adults. It can be divided into hemorrhagic stroke (~15%) and ischemic stroke (~80%) 1. Actively exploring various treatment methods to promote the prevention of stroke high-risk groups and rehabilitation of the nerves post-stroke is the key to save stroke patients. With the development of global aging, the development of new potential therapies for neurodegenerative diseases is also the prerequisite for improving quality of life among the elderly and reducing the heavy burden of public management and caregivers 2. The clinical transformation of preparations based on nanotechnology brings new hope for drug selection and targeted delivery. It is expected to be able to effectively overcome the CNS obstacles and transfer drug molecules to the brain parenchyma. Traditional drug delivery systems (DDSs), including polymeric nanoparticles (NPs) 3, solid lipid NPs 4, nanoemulsions 5, and liposomes *etc*. 6, have been used as effective tools to target the brain 7, 8. The development of cellular-specific therapy also opens new possibilities for the treatment of neurological diseases, but the use of living cells may induce an autoimmune response or rejection, which may increase the risk of sustained stimulation. Considering the complexity of production process, safety and biocompatibility, naturally occurring nanovesicles are superior in this aspect.

Exosomes were first discovered in reticulocytes through electron microscopy analyses in 1983 9. Since then, studies have found that exosomes can be secreted by almost all mammalian cell types. Therefore, exosomes are captured in primary cells of the immune and nervous system, stem cells, many cancer cell lines, and in blood, tears, urine, saliva, milk, and ascites among other body fluids 10. In addition, exosomes are also found in lower eukaryotes and prokaryotes. Recent studies have proven that plant-derived exosome-like nanoparticles (ELNs), which are called botanosomes, can also be extracted from herbs, such as *Rhodiola rosea L.* (Crassulaceae) and *Dandelion* (Taraxacum officinale Weber) 11.

Although exosomes have been known for 30 years, their biology and functions are still not fully understood. As extracellular vesicles, exosomes are membrane nanovesicles with a diameter of 40-150 nm that contain proteins, RNAs, DNAs, lipids, metabolites, and cytosolic molecules 12. Therefore, exosomes have abundant functions, such as realizing intercellular communication, transferring antigens to dendritic cells (DCs), promoting angiogenesis and achieving homing targeting 13. For instance, the high expression of transmembrane proteins (especially CD9, CD63 and CD81) and miRNAs, such as miR-124, miR-132 and miR-212, has been confirmed to give exosome targeting functions 14. In the nervous system, exosomes help promote myelination, neurite outgrowth, and neuron survival and stimulate tissue repair and regeneration 15, 16. They have evolved from simple “cellular trashcans” to key roles in many biological processes under pathological and nonpathological conditions. In particular, exosomes from different sources can be transported across the blood-brain barrier (BBB) in various ways 17, 18.

Based on the structure and function of the exosomes mentioned above, exosomes have become popular in the treatment of cerebrovascular and neurodegenerative diseases. A previous study indicated that systemic administration of mesenchymal stem cell-derived exosomes (MSC-Exos) post stroke could improve functional recovery and enhance angiogenesis, neurite remodeling and neurogenesis 19. Another study proposed that MSC-Exos reduced inflammation and prevented abnormal neurogenesis by acting on astrocytes, showing great potential in the treatment of neurological diseases, such as stroke, temporal lobe epilepsy, Alzheimer's disease, and Parkinson's disease 20.

Although the homing function of exosomes has great potential in treatment, it has been verified that their targeting ability is inefficient in animal experiments 21. Therefore, a large number of engineering technologies have been used to modify exosomes to enhance their targeting ability. Another potential application of exosomes is monitoring capabilities in clinical practice. Since the membrane structure of exosomes can be easily labeled and exosome markers are tightly connected, changes in biomarkers can be monitored when the patient receives effective treatment 22. The specificity of exosomes makes them have different half-lives and distributions *in vivo* 23, 24, which confirms the vital role of exosome biomarkers in tracking exosome fate. In addition, exosomes are involved in complex information exchange between cells, thus regulating various processes (e.g., homeostasis, angiogenesis and immune response) under pathological and physiological conditions. Tracing exosomes *in vivo* can help us understand their mechanism of action, biological function, biological distribution, migration ability, and communication ability. At present, as exosomes have become popular nanocarriers for development in drug delivery systems, the route of administration is also an important point that should not be ignored 25.

**Figure 1:**
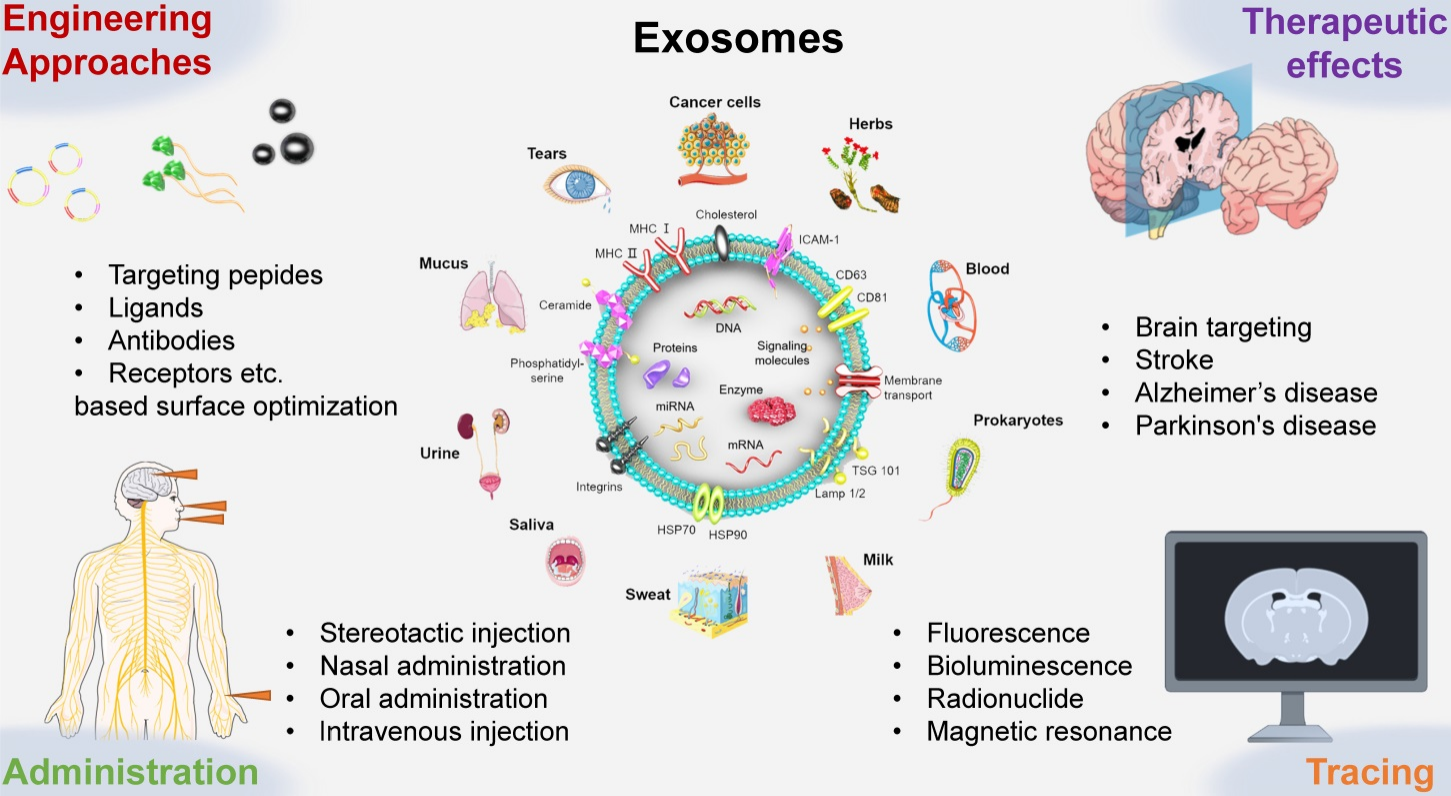
Schematic presentation of the use of engineered exosomes as novel therapeutic tools for brain targeting against cerebrovascular and neurodegenerative diseases. Exosomes can be extracted from a wide range of sources, including immune and cancer cell lines, blood, tears, urine, saliva, milk, ascites, eukaryotes, prokaryotes, herbs, et al. This review mainly introduces the administration routes, engineering targeting strategy, therapeutic effects, labeling and tracking methods of exosomes.

In this review, we summarized the administration routes, engineering strategies, targeting efficiency, therapeutic effects and tracing methods of exosomes (Figure 1). Moreover, we also analyzed the internalization and special regulatory effects of exosomes on the BBB. This review can improve our understanding of the biological significance of exosomes and thus promote significant progress in the treatment of cerebrovascular and neurodegenerative diseases.

## Administration routes of exosomes

Despite significant progress in separation and purification, the effective delivery of exosomes to the brain remains a major challenge. External therapeutic exosomes can be easily recognized by the immune system and captured by the reticuloendothelial system, but the main hurdle of efficient drug delivery to the central nervous system (CNS) is passing through the BBB 26, 27. In addition to the mentioned characteristics of exosome, routes of administration also have an important influence on the biodistribution, therapeutic effect and short-term/long-term biological effects of exosomes. The main administration methods for cerebrovascular and neurodegenerative disease therapies include intravenous injection, oral administration, stereotactic injection, and nasal administration (Table 1).

### Intravenous injection

Intravenous injection is the most widely used route for exosome administration 48. In early 2010, Lai RC et al. protected the heart function of mice by intravenous injection of exosomes, which significantly reduced the infarct area of MIR injury in the mouse model by approximately 50%. The exosomes were derived from MSCs (MSCs-Ex) in a mouse model of myocardial ischemia-reperfusion injury 49. Xin H et al. found that intravenous injection of MSCs-Ex could intensify the PI3K/Akt/mTOR/GSK-3β signaling pathway by targeting PTEN, increasing nerve differentiation and plasticity, and improving nerve function after stroke 50. Zhang H et al. achieved targeted delivery of exosome-mediated miR-210 by intravenous injection. The results of upregulation of VEGF and CD34 indicate improved angiogenesis and a significant increase in animal survival 31. Cui GH et al. found that intravenously injected exosomes could be tracked in the brain and proved to significantly improve learning and memory abilities, reduce plaque deposition and Aβ levels, and normalize inflammatory cytokine levels 51. In a mouse Parkinson's disease model, exosomes containing catalase were successfully delivered to the brain by intravenous injection to protect SNpc neurons in mice with brain inflammation from acute oxidative stress 40. Although intravenous injection is a widely used method, the half-life index of systemically administered exosomes is still too short, ranging from several minutes to a few hours. In addition, the blood circulation of exosomes is easily cleared by macrophages 52, 53.

### Oral administration

Despite considerable progress in the route of intravenous injection, oral administration is also a vital delivery route in clinical practice. The main significant advantages of oral administration are 1) less fluctuation of drug levels in plasma, 2) good patient compliance and acceptance, and 3) low cost-effectiveness. In addition, exosomes for oral drug delivery systems can solve the following problems: 1) the solubility of drugs and 2) the reduction of therapeutic indicators and toxicity compared to free drugs 54, 55. For example, Nazimek K et al. found that miRNA-150-carrying exosomes from suppressor T cells and B1a cells could effectively induce long-term inhibition of delayed-type hypersensitivity (DTH) after a single systemic administration in mice. In this study, they compared the therapeutic effects of OVA-specific suppressor T cell-derived exosomes via intravenous (iv), intraperitoneal (ip), intradermal (id) and oral administration in active mice immunized with OVA DTH. All of these administration routes could significantly inhibit DTH ear swelling, and the strongest effect was observed after oral treatment 56. Aqil F et al. utilized milk-derived exosomes to load curcumin to obtain exosomal curcumin (ExoCUR). Compared with free curcumin, it was found that the oral administration of ExoCUR could increase the drug content by 3-5 times 57. Although oral administration may have many advantages, there are still some major hurdles in the application of exosomes, such as poor bioavailability and first pass metabolism in the small intestine. Most of the exosomes were localized in the small intestine, the unabsorbed part was transferred to the colon, a small part (<5%) was diffused in the liver, and exosomes was difficult to spread to the brain. In addition, the effects of microorganisms and enzyme digestion in gastrointestinal fluid on the structural integrity of exosomes also need to be considered 58. The exosomes used for oral administration in current research are still limited to food-derived exosomes, especially those derived from milk 59. After purification, the amount of exosomes derived from non-food sources, such as secreted by cells, is not enough to meet oral requirements. In research of Record M, 2 mg of exosomal preparation were oral delivered to mice daily for 3 weeks, accordingly, a person weighing 70 kg required about 150 g of these preparations 60.

### Stereotactic injection

Stereotactic injection refers to dissolving or dispersing the drug and polymer in an appropriate solvent and injecting it locally near the target site 61. Stereotactic injection not only solves the problems of poor stability, off-target toxicity, and poor drug release repeatability but also reduces the retention time, the dose, and the toxicity and side effects of the drug 62. Orefice NS et al. monitored the *in vivo* spreading of exosomes after stereotactic administration in real time 63. In detail, the unassociated adeno-associated virus (std-AAVs) encoding GFP were encapsulated in HEK293 cells-secreted exosomes and injected into ipsilateral hippocampus of mice. The results indicated that the use of exosomes had a greater distribution of GFP from the injection site to the contralateral side, while GFP was mainly located in the ipsilateral hemisphere without exosomes. In addition, it was also observed that the AAV mediated fluorescence from the injection site using exosomes could last up to 95 days after injection, compared to 31 days without exosomes. Yuyama K et al. injected biotinylated exosomes into the right hippocampus of PP_SweInd_ transgenic (APP) mice using stereotaxic coordinates 64. The results confirmed that Aβ was captured on the surface of exosomes and transported to microglia for degradation. Infusion of exosomes into the brain led to a decrease in Aβ levels, improved Aβ-related pathology in APP mice, and further provided a novel strategy for the treatment of Alzheimer's disease. In terms of stroke recovery, Gao B et al. investigated the biological function and mechanism of cerebral endothelial cell-derived exosomes (ECs-exo) on brain plasticity 65. After intravenous injection, the exosomes quickly disappeared from the systemic circulation of mice by a rapid clearance phase through liver and kidney pathways within 6 h. On this basis, they stereotaxically injected ECs-exo into the ventricle of rat's brain to achieve better accumulate around the lesion, thereby exerting long-term effects. ECs-exo containing microRNA-126-3p upregulated gene expression related to plasticity signal, changed neural plasticity of motor cortex, and positively regulated synaptic plasticity during stroke recovery. This study suggested that ECs-exo intraventricular injection promoted functional motor recovery in the middle cerebral artery occlusion (MCAO)/R model and played a crucial role in synaptic function reconstruction in ischemic brain injury. Besides, Zhou ST et al. demonstrated that exosomes isolated from bEnd.3 cells by ultracentrifugation could attenuate infarct volume, activate the proliferation and migration of neural progenitor cells, and promote neurogenesis in periinfarct area, subregion of subventricular zone (SVZ) and dentate gyrus (DG) area of hippocampus 43. Although stereotactic brain injection is the most direct and accurate route to transport exosomes to the target structure, it still faces many difficulties. Stereotactic injection has high requirements for equipments and operative skills, and imaging guidance is also needed to locate the target position. Injection deviation may cause serious brain damage. Moreover, anesthesia and surgical procedures may cause severe wound pain, infection, procedural complications, or even neurological problems 66.

### Nasal administration

Nasal administration is a promising option that bypasses the BBB for the CNS drug delivery 67. The two main routes of intranasal drug absorption are the olfactory and trigeminal nerve pathways, which can bypass the BBB and the blood-cerebrospinal fluid barrier, and transporter regulation can also help nasal delivery of its substrate 68, 69. In the study of brain diseases, compared with intravenous injection, nasal administration could more effectively transport exosomes to the brain 70. Nasal exosome administration has attracted widespread attention because this process is noninvasive, suitable for repeated distribution, and leads to rapid penetration of exosomes into multiple areas of the forebrain 71. Haney MJ studied the transport of exosomes into mouse model with 6-OHDA-induced brain inflammation through both intranasal and intravenous routes 40. The results showed that exosomes were widely distributed in the whole brain of mice, mainly in the cerebral frontal cortex, cerebellum central, and sulcus after intranasal administration. Perets N et al. delivered gold nanoparticle (GNP)-labeled MSC-exos nasally, and X-ray computed tomography (CT) technology was used to track the migration and homing of different brain diseases, including stroke, Alzheimer's disease and Parkinson's disease 72. As nasal administration could bypass the BBB and directly enter the brain, it was found that MSC-exos stayed in the brain for up to 96 h tracking noninvasively on CT. The authors also demonstrated that the pathology-specific homing pattern of MSC-exos was significantly related to pathology-related neuroinflammation. Long Q et al. found that administering MSC-derived A1 exosomes through the nasal cavity could reduce inflammation and prevent memory dysfunction or abnormal neurogenesis after status epilepticus 73. Kalani A et al. used nasal delivery of curcumin-loaded embryonic stem cell exosomes (MSC-exo-cur) to restore neurovascular units after ischemia-reperfusion injury 74. Compared with untreated IR-injured mice, MSC-exo-cur treatment restored vascular endothelial tightness (claudin-5 and occludin) and the adhesion protein (VE-cadherin) connexin. However, intramural administration also has some disadvantages, including relatively small dosage, limited surface area of olfactory epithelium, short retention time of drug absorption and its effect on nasal secretions 67, 75.

In addition to the above common routes of administration, the researchers also tried some novel injection locations. Zhao P et al. injected indocyanine green (ICG) PLGA nanoparticles into the neck near the local lymph nodes by subcutaneous injection and found more than 44 times more absorbed in the brain than by the intravenous route 76. This method can further improve the bioavailability of the drug, prolong the action time of the drug, and realize the accumulation of the drug in the disease sites.

## The ability of exosomes to cross and to regulate the blood-brain barrier (BBB)

**Figure 2:**
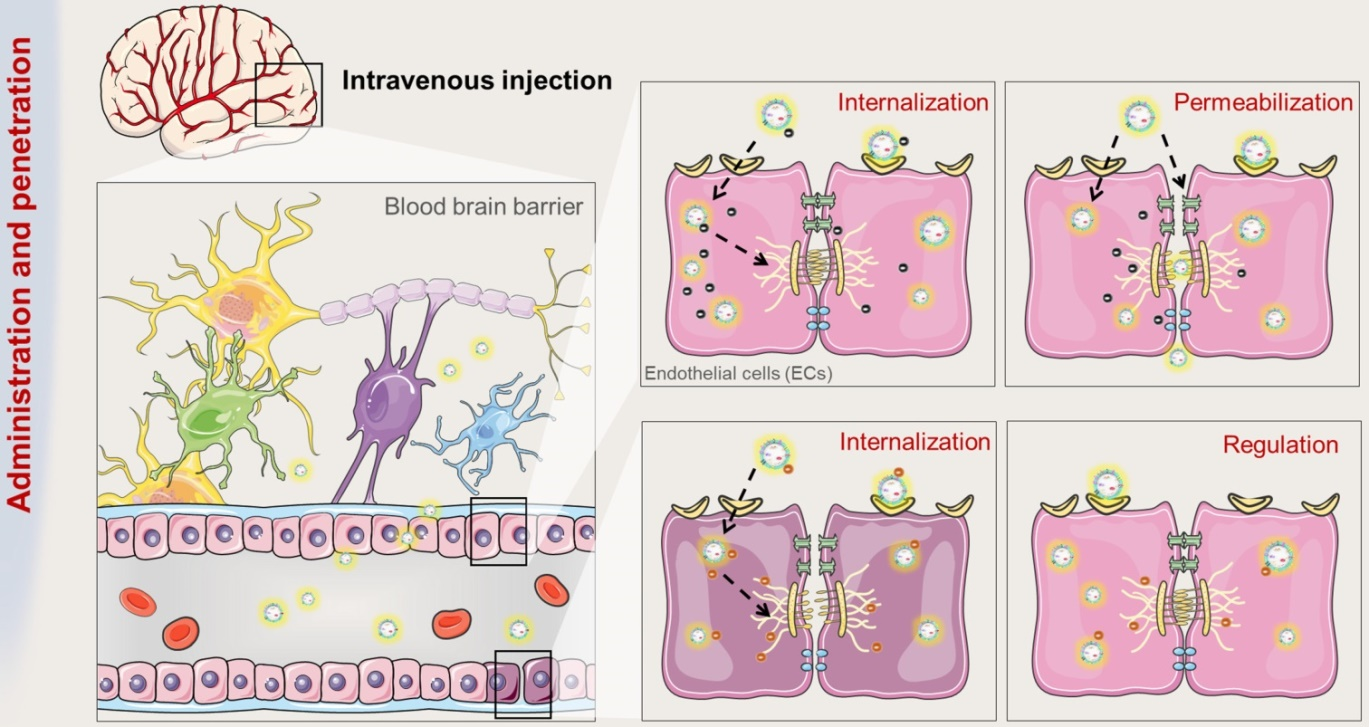
The route and mechanism by which exosomes transport across BBB. Brain microvessels endothelial cells (ECs) are the bricks supporting the BBB phenotype. On the one hand, exosomes can be internalized by ECs and penetrate the BBB. On the other hand, the internalization of exosomes can improve the integrity of BBB.

Despite significant advances in DDSs, there are still major challenges in effectively delivering drugs to the brain for the treatment of CNS diseases, one of which is the limitation by the presence of BBB. At present, intravenous injection is still the most widely used administration route, which requires that drug carriers can be directly transported across the BBB and further evenly distributed through brain capillaries 77. It has been proven that exosomes can promote intercellular communication between adjacent cells or even distant organs by carrying proteins and RNAs (Figure 2) 78, 79. Using zebrafish as an animal mode, Yang T et al. evaluated the exosome-mediated delivery across BBB and explained the transport mechanisms 80. In this study, zebrafish embryos were injected with rhodamine 123-loaded exosomes *via* the cardinal vein, and fluorescence of rhodamine 123 was examined in the circulating system *in vivo*. When given alone, rhodamine 123 remained within the blood vessels and was not observed in the brain tissue. In contrast, when delivered by brain endothelial bEND.3 cells-derived exosomes, rhodamine 123 showed significant penetration in brain regions, confirming the ability of exosomes to deliver drugs across BBB for brain diseases treatment.

### Barrier function of endothelial cells in BBB

The development of the cerebrovascular system, including its integrity, is strictly regulated by peripheral nerve tissue. The BBB is located at the interface between CNS and peripheral circulation system. As a highly selective membrane, BBB possessed ability to restrict the exchange of substances between the brain and blood vessels to maintain brain's homeostasis and protect the brain's microenvironment from exogenous sources and circulatory system threats. For a long time, crossing the BBB remains a major obstacle to the development of new brain therapies 81, 82. In the strong wall of the cerebral microvascular system constructed by the BBB, cerebral microvascular endothelial cells (ECs) are bricks supporting the BBB phenotype 83. The characteristics of ECs determine the characteristics of the BBB. ECs connect with each other through junction proteins, and the tight junctions (TJs) between endothelial cells block the paracellular pathway. The barrier function of ECs mainly comes from the following two components: 1) the metabolic barrier supported by enzymes and efflux pumps (e.g., P-glycoprotein and multidrug-resistance proteins (MRPs)) that limits nonspecific transport and facilitates receptor- or transporter-specific pathways and 2) the physical barrier that limits the intercellular action of cells through apical TJs and closes the paracellular space between ECs 84.

### Endocytosis of exosomes by endothelial cells

After being released into the extracellular space, exosomes can be internalized by recipient cells through different mechanisms, such as endocytosis, micropytosis, phagocytosis, and plasma membrane fusion 85. Many current studies on how exosomes cross the BBB have pointed out that transcellular transport is carried out through ECs by endocytosis-based uptake 79. Chen CC et al. simulated both healthy and inflammatory BBB conditions *in vivo* and studied the interactions between exosomes and BMECs *in vitro* 86. The results showed that luciferase-carrying exosomes (hGluc-Lact exosomes) could be internalized by BMECs and more effectively crossed the BMEC monolayer under stroke-like conditions. Exosomes were internalized by BMECs through endocytosis and accumulated in endosomes. In addition, most exosomes crossed the BMEC monolayers *via* a transcellular pathway (i.e., endocytosis on the other side of the translayer, MVB formation and exocytosis) while only a small number of exosomes pass through the paracellular route (i.e., passive diffusion though intercellular space between BMECs). Another study by Yuan D et al. verified the uptake of Mϕ-derived exosomes (Mϕ-EXO) in human brain microvascular ECs 23. The results of endocytosis pathway analysis showed that Mϕ-EXO could enter ECs through clathrin, pits and macropinocytosis. The colocalization of Mϕ-EXO with anti-clathrin heavy chain antibodies, transferrin, anti-caveolin 1 antibodies and CTB further supported the participation of caveolin and reticulin pathways. The authors further verified that Mϕ-EXO inherited LFA-1 from their parental cells. This protein interacted with endothelial ICAM-1 and mediated the lateral migration of Mϕ-EXO on BBB. Generally, the uptake of exosomes in BMECs may depend on specific lipid rafts or ligand receptors, and the mechanism of uptake of exosomes varies with different cell sources. Exosomes secreted by different cells may have different cargo, including proteins and lipids, which may change the way they cross BBB 87, 88. In addition, the disease state may also affect the methods of BBB crossing, as the cargo may change with the disease state.

### Effect of exosomes on BBB integrity

Once the exosomes are internalized by recipient cells, the cargo they carried may elicit an effect on the recipient cells, leading to changes in the barrier's properties 27. In addition to endocytosis, it has been proved that the destruction of BBB could be triggered by exosomes through another mechanism. Recent studies have clarified the role of exosomes in increasing the permeability of BBB. Zhou W et al. found that exosomes derived from breast cancer cells could specifically express miR-105, which could directly target the TJ protein ZO-1 (a central molecular component of TJs) and reduce the integrity of the BBB 89. In addition to exosomes secreted from diseased cells, a similar phenomenon was also found in exosomes from normal cells. Pivoraitė U et al. demonstrated for the first time that exosomes secreted by human dental pulp stem cells (DPSCs) could increase vascular permeability in the initial stage of acute inflammation and then suppress carrageenan-induced acute inflammation in mice model 90. It should be noted that this endocytosis-associated TJ remodeling might be transient and reversible 91. Therefore, the route by which exosomes enter tissues may be related to the permeability of the vascular endothelial barrier. Furthermore, central nervous system diseases, such as cerebrovascular diseases, often cause drastic changes in the structure and function of the BBB 92. The permeability of the barrier is significantly improved so that macromolecular substances such as plasma albumin can pass through the barrier. Severe brain injury leads to serious damage of the BBB, allowing serum proteins to enter the brain tissue through the barrier, posing a great threat to the life of patients. For this reason, researchers have also explored the role of exosomes in the BBB of nervous system disease models. Xu B et al. examined whether neurons secreting miR-132-containing exosomes could regulate the brain vascular integrity 93. Using cultured rodent brain cells and intact zebrafish larvae, the researchers found that neurons maintain the integrity of the BBB by secreting exosomes into ECs, thus transferring miR-132 (a highly conserved and neuron-enriched microRNA). After internalization of exosomes into ECs, miR-132 could directly target eukaryotic elongation factor 2 kinase (eef2k), thereby regulating the expression of vascular endothelial cadherin (an important adherens junction protein). This study revealed previously unidentified functions of miR-132 and revealed that exosomes could be new vehicles for mediating cerebrovascular integrity and treating neurological diseases. In addition to miR-132, other factors may also be involved in the neuroregulation of cerebrovascular integrity 94. Gao WW et al. demonstrated that exosomes secreted by human umbilical cord blood-derived endothelial colony forming cells (ECFCs) could restore the continuity of damaged BBB in mice 95. These exosomes were highly absorbed by ECs, subsequently promoted the migration of ECs, inhibited PTEN expression, and increased AKT phosphorylation and TJ protein expression in hypoxia-exposed ECs. After intravenous administration, exosome treatment reduced the permeability of BBB and improved the recovery of neurological function post-injury. All these studies indicate that exosomes secreted by different cell sources can affect vascular permeability, and in some cases, affect the integrity of BBB, thus playing a regulatory role in the homeostasis of the BBB.

## Engineering strategies of exosomes for targeted brain delivery

As exosomes are released during the fusion of multivesicular bodies (MVBs) with the plasma membrane, specific miRNAs, mRNAs, and proteins can be detected in exosomes derived from various cells. The characteristics of exosomes that promote the ideal delivery of biopharmaceuticals include their ability to cross biological membranes or barriers to efficiently transfer their payloads to recipient cells, as well as their low immunogenicity, noncytotoxicity, and good circulation stability 24, 96.

**Figure 3:**
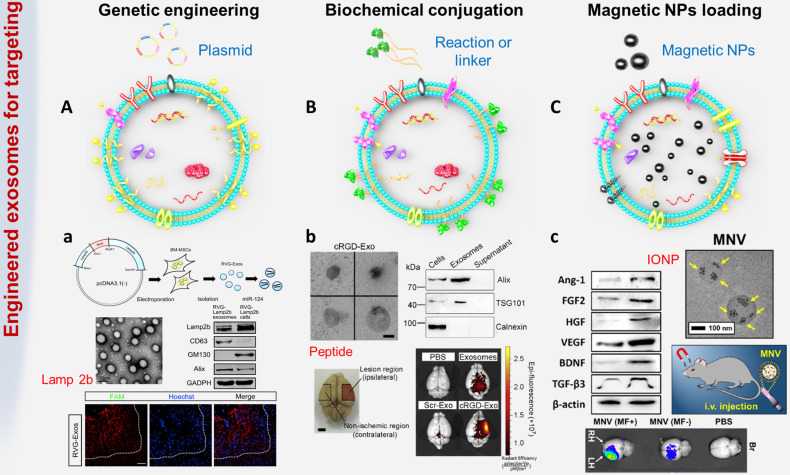
Common exosome engineering strategies. **(A)** Genetic engineering: the foreign gene is transferred into the target cell by plasmid, so that the exosomes can carry specific targeted proteins or peptides. a. BM-MSCs were genetically engineered to secrete exosomes expressing rabies virus glycoprotein (RVG) fused Lamp2b to achieve targeted delivery of gene drugs to the brain (adapted with permission from 29, copyright 2017 Elsevier). **(B)** Biochemical engineering: using biochemical conjugation or hydrophobic insertion or chemical coupling to conjugate functional ligands to exosomes. b. The surface of MSC-derived exosomes was conjugated with targeting peptide through bio-orthogonal chemistry method (adapted with permission from 19, copyright 2018 Elsevier). **(C)** Physical targeting: loading magnetic particles to make exosomes accumulate at the target site. c. Magnetic nanovesicles (MNV) containing iron oxide nanoparticles (IONP) were prepared to achieve magnetic navigation mediated accumulation (adapted with permission from 104, copyright 2020 Elsevier).

Due to the complex pathogenesis of brain diseases, the lack of effective and specific recognition epitopes greatly hinders the clinical application of exosomes 97, 98. Although the complete transmembrane proteins (CD81 and CD9) and integrins (CD51 and CD61) in exosomes are considered to have certain homing and targeting functions, their targeting ability is still too weak to be applied 99. To improve the targeted delivery efficiency of exosomes in the treatment of brain diseases, optimization of exosomes by engineering technology has become an effective method. Currently, most of the targeted methods aim to use specific ligand/receptor binding strategies to promote the coupling of exosomes with target cells, thereby promoting endocytosis 100. Studies have shown that certain molecules on the surface of stem cells can play a role in the targeting ability of exosomes. Stem cell-derived exosomes can be delivered to subgranular areas of the hippocampus associated with mood disorders to improve neurogenesis. Raposo G et al. found that the surface protein composition of exosomes was significantly different from that of the plasma membrane, as Class II MHCs bound by exosomes were in a tight peptide-bound conformation. Therefore, exosomes secreted by B lymphocytes could bind antigen-specific class II MHC to inhibit the activation of T cells, indicating the importance of exosomes in the process of antigen presentation *in vivo* 101, 102. In addition, Ohno S et al. found that the GE11 peptide had high affinity for epidermal growth factor receptor (EGFR), a protein abundant in a variety of human epithelial tumors. Exosomes containing GE11 peptides and loaded with miRNA let-7 could fuse with the transmembrane domain of platelet-derived growth factor (PDGF) receptor to inhibit tumor growth *in vivo* 103. The feasibility of targeting exosomes to specific cell types will open the door to the treatment of cerebrovascular and neurodegenerative diseases. Inspired by this possibility, an increasing number of studies have focused on exploiting multiple strategies to engineer natural exosomes to achieve a better target rate (Figure 3).

### Genetic engineering

Genetic engineering technology is a complex technology for manipulating genes at the molecular level, and it can transfer foreign genes into recipient cells through *in vitro* recombination so that the genes can be copied, transcribed and expressed in receptor cells 105. Studies have confirmed that certain proteins can interact with specific cell receptors or extracellular matrix components expressed in cells of the cerebrovascular system 106, 107. To endow exosomes with stronger targeting properties, the genes expressing these targeted proteins or peptides were transferred into recipient cells, and then the secreted exosomes carried specific homing proteins or peptides, such as HER2 and Lamp2 (Figure 3A).

The most common method is to use genetic engineering technology to make recipient cells highly express targeting proteins or polypeptides, thereby secreting target-specific exosomes. For example, Liang G et al. fused HER2 with the extracellular N-terminus of human Lamp2, a protein found in a large number of extracellular membranes, and then cloned it into pLVX-GFP-N1 to obtain the final fusion protein called THLG 108. Then, HEK293T cells were stably transduced with a lentiviral vector encoding THLG. Exosomes with enhanced targeting function were purified and collected from the culture supernatant of THLG-293T cells by ultracentrifugation. Experimental results found that using THLG as a ligand for HER2 could significantly enhance the binding ability of exosomes to target cells, thereby effectively reversing drug resistance and improving the efficiency of cancer treatment. Alvarez-Erviti L et al. transfected a plasmid encoding Lamp2b (exosomal membrane protein fused with neuron-specific RVG peptide) into dendritic cells through genetic engineering 37. Through intravenous injection, RVG-targeting exosomes could be specifically delivered to neurons, oligodendrocytes and microglia of wild-type mice, leading to specific gene knockdown. The powerful protein (62%) and mRNA (60%) inhibition of BACE1 (a therapeutic target for Alzheimer's disease) demonstrated the therapeutic potential of exosome-mediated siRNA. Exosomes obtained by genetic engineering have been confirmed to achieve the expected transformation and improve targeting ability both *in vitro* and *in vivo*. However, these engineering processes are complex and costly, and most importantly, this method cannot be applied to the pre-separated exosomes or the natural exosomes existing in body fluids.

### Biochemical engineering

Biochemical engineering is another exciting technique used to improve the targeting of exosomes (Figure 3B). Compared with genetic engineering, using biochemical engineering to modify exosomes is simpler, faster, and more effective without modification of cells. The first strategy is direct modification through membrane fusion or hydrophobic insertion. The surface properties of exosomes can be easily fused with liposomes embedded with peptides or antibodies as targeting moieties. For example, Matsuoka T et al. obtained exosomes from cells expressing the tyrosine kinase receptor HER2, and next fused these HER2-containing exosomes with phospholipid liposomes by the freeze-thaw method 109. This strategy not only optimized the surface characteristics of exosomes, thereby reducing their immunogenicity and increasing their stability, but also prolonged the half-life of exosomes in the blood. In addition, Kooijmans SAA et al. proposed a 'postinsertion' mechanism as a new technology to endow exosomes with targeting capabilities 110. This method used a targeting ligand conjugated with polyethylene glycol (PEG) to modify exosomes. Researchers first prepared nanobody-PEG micelles by combining epidermal growth factor receptor (EGFR) specific nanobodies with phospholipid (DMPE)-PEG derivatives. When these micelles were mixed with exosomes derived from Neuro2A cells or platelets, the temperature-dependent transfer of nanobody PEG-lipid to the exosome membrane could be observed. This process did not affect the morphology, size distribution or protein composition of electric vehicles. More importantly, the insertion of ligand-conjugated PEG-derived phospholipids in the exosome membrane allowed the exosomes to have higher cell specificity and longer circulation time, which might increase the accumulation of exosomes in target tissues and improve cargo transport efficiency. The second strategy is using chemical methods to conjugate functional ligands to the surface of exosomes. For instance, Cui GH et al. synthesized the system-specific rabies virus glycoprotein (RVG) peptide and then coupled it to exosomes using the G protein coupling method 51. In detail, DOPE-NHS (dioleoylphosphatidylethanolamine N-hydroxysuccinimide) was reacted with the RVG peptide to obtain DOPE-RVG, which was subsequently incubated with MSC-derived exosomes. Compared with MSC-Exos, the plaque deposition and Aβ level of the MSC-RVG-Exo-treated group dropped sharply, and the activation of astrocytes was significantly reduced. Functional exosomes efficiently accumulated in the brains of transgenic APP/PS1 mice. In addition, Tian T et al. proposed a rapid and easy method using the bioorthogonal copper-free azide alkyne cycloaddition method (click chemistry) to modify the surface of mesenchymal stromal cell (MSC)-derived exosomes 21. The functional ligand cyclo(Arg-Gly-Asp-D-Tyr-Lys) peptide [c(RGDyK)], which has a specific high affinity for integrin α_v_β_3_ in reactive cerebral vascular endothelial cells after ischemia 104, 111, was efficiently conjugated to achieve the desired exosome modification. Compared with free curcumin or exosomal treatment alone, cRGD-Exo containing curcumin (cRGD-Exo-cur) was more effective in suppressing the inflammatory response and cellular apoptosis in the lesion area, indicating the actively targeting ability of engineered exosomes to reactive cerebral vascular endothelial cells after ischemia. One major advantage of directly modifying exosomes without changes at the cellular level is that reagents and reaction conditions that cannot be used to functionalize living cells can be utilized. Nevertheless, in the process of biochemical engineering, it is necessary to consider that temperature, pressure, and salt concentration may cause excessive osmotic pressure, membrane rupture and surface protein denaturation.

### Physical engineering

Biochemical engineering requires multiple chemical reaction steps, which may affect the function of exosomal proteins. The stability of surface modification highly depends on the bonding strength between the exosomes and exogenous species. In the complex cycle of the internal environment, disruption of the connection part may lead to off-target effects on exosomes. Because of this effect, studies have explored using physical methods, such as coincubation, ultrasound, and electroporation, to stimulate exosomes to load targeted functional nanomaterials 112. These methods cause little damage to exosomes and have little effect on their function.

One of the feasible strategies is to pack exogenous substances into the cavity of exosomes instead of surface modification (Figure 3C). Kim HY et al. incubated iron oxide nanoparticles (IONPs) with mesenchymal stem cells (MSCs) to obtain magnetic nanovesicles (MNVs), which were also known as theranosomes with potential for *in vivo* magnetic targeting 113. Under the guidance of an external magnetic field (MF), MNVs can be magnetically navigated to target ischemic lesions in the brain. In the transient MCAO-induced rat model, the specific accumulation of MNV after systemic injection was 5.1 times higher than that of the control group. Furthermore, IONPs could stimulate the expression of therapeutic growth factors in MSCs by activating the phosphorylation of c-Jun and c-Jun N-terminal kinase (JNK) molecules. IONPs are also biocompatible as they can be assimilated into iron ions and ferritin. 114.

### The choice of strategy for appropriate modification of exosomes

The abovementioned methods have both advantages and drawbacks. Genetic methods are more accurate in obtaining the desired products, but the process is complicated and costly. Biochemical methods can effectively control the functional structure of the exosome surface (including unnatural amino acids that prevent peptide degradation) and the density of targeted epitopes, but the reactions require multiple biochemical steps, which may impair the function of carbohydrates and proteins on the exosome membrane. Physical targeting is a noninvasive method with less damage to exosomes, but complex auxiliary equipment is required for the preparation process or *in vivo* guided targeting.

All these engineering strategies provide attractive prospects for expanding the targeting efficiency and therapeutic capacity of exosomes beyond their original function. When selecting an appropriate engineering method, it is necessary to consider the source cell type, the cargo to be loaded, the disease to be cured, and other related factors. Moreover, exosomes also need to be fully characterized and detected after engineering to confirm their morphology, surface composition and stability. In addition to the study of cell uptake of exosomes, we should further explore the mechanism of the interaction between exosomes and receptor cells after membrane modification and the release of the cargoes loaded by the carriers. In the process of engineering, if some of the original surface ligands of exosomes are inactivated, their corresponding signal cascades may not be triggered, thus reducing the internalization of exosomes in receptor cells. Changes in exosome surface charge, membrane rigidity and immunogenicity will also affect exosome stability, *in vivo* fate and follow-up therapeutic effects. The brain-targeted engineered exosomes involved in recent studies are summarized in Table 2.

## Engineered exosomes as smart nanoscale therapeutics

**Figure 4:**
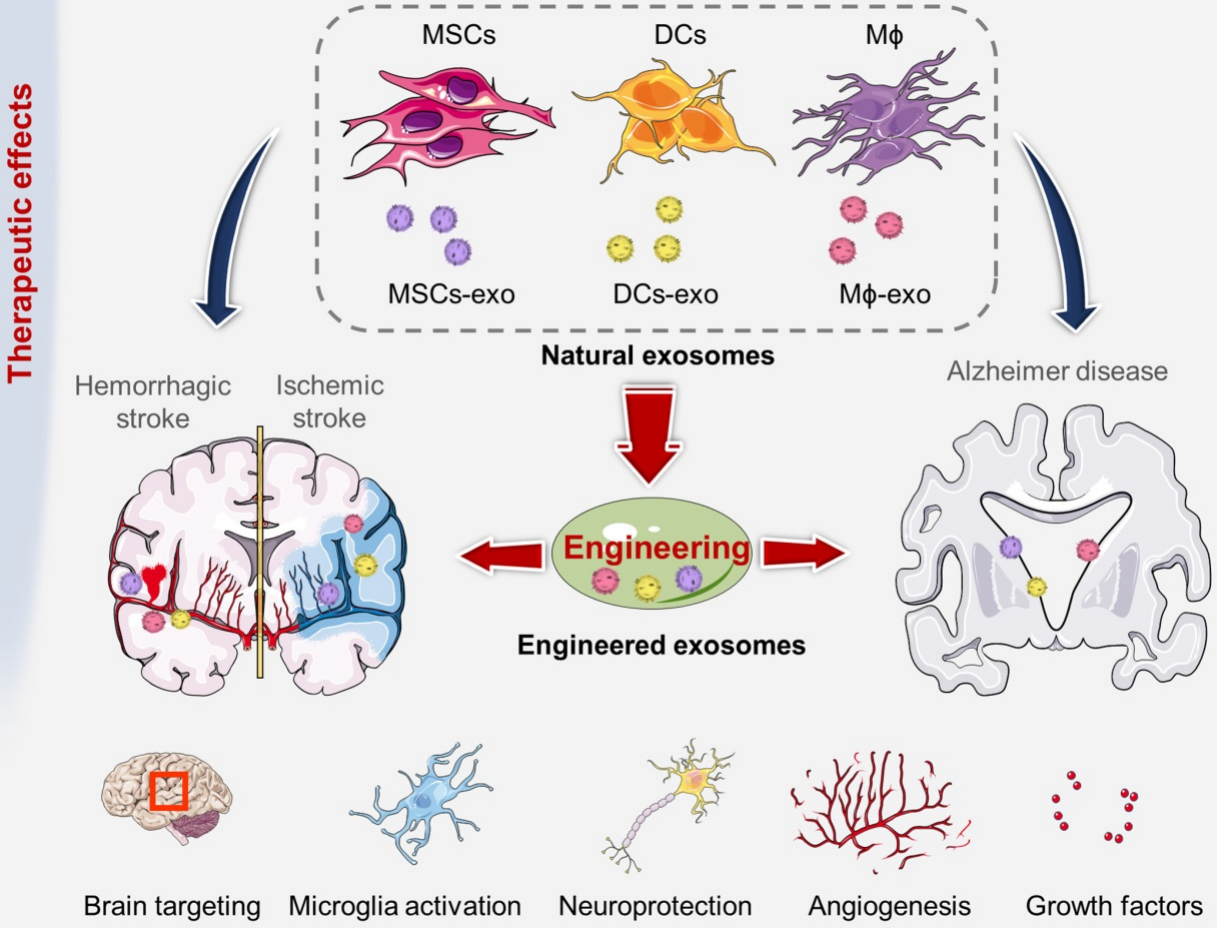
The therapeutic role of exosomes derived from various cell sources have been used to treat cerebrovascular and neurodegenerative diseases.

Recent studies have shown that exosomes participate in long-distance intercellular communication and promote the transfer of proteins, miRNAs and functional mRNA for subsequent protein expression in target cells 119. As intercellular communication messengers of cerebrovascular and neurodegenerative diseases, exosomes could be applied as both diagnostic and therapeutic tools. After allogeneic administration *in vivo*, exosomes will not block blood vessels or cause significant adverse reactions 120. Compared to direct delivery of living cells, exosomes have the ability to cross the BBB easily, which shows unique advantages in the treatment of brain diseases 121. In addition, exosomes can be stored at -80 °C for 6 months without degradation, which can effectively protect internal soluble molecules, including biological factors and nucleic acids 122. Although unmodified natural exosomes have targeting ability, they still have the problems of low targeting efficiency, short circulating half-life and poor efficacy. Various engineering methods can be used to modify homing peptides or ligands on the surface of exosomes, which can endow exosomes with targeting ability and thus improve their therapeutic efficiency (Figure 4).

### Promising cellular sources of exosomes

The molecular characteristics and regulatory function of exosomes mainly depend on the types of cells that secrete them. Among all exosome-based treatments for brain pathologies, the most promising cellular sources are dendritic cells (DCs), human mesenchymal stem cells (MSCs), and macrophages (Mϕ). Presently, MSCs are a widely used cell type in clinical trials because of their reported regenerative potency and differentiation potential, which is being reported to reduce neurological deficits after stroke 123. Several studies have shown that MSC-derived exosomes (MSC-exo) retain important characteristics of their parental MSCs, such as neurite remodeling, immune modulation, angiogenesis promotion, and notably, migration and homing abilities 124, 125. Perets N et al. determined that MSC-exo presented distinct brain migration and homing patterns in various brain pathologies after nasal administration 72. In a focal ischemic stroke model (*via* ETH1-1 injection), MSC-exo were mainly distributed in the injection area of the striatum. In the Parkinson's disease model (6-OHDA induced) 126, a large number of MSC-exo migrated to the striatum, as well as to the midbrain and cerebellum. In an Alzheimer's disease model (transgenic 5xFAD mice), MSC-exo were found in the hippocampus, which was the central region related to this disease 127. In contrast, in healthy brains, MSC-exo showed diffuse migration patterns and clearance within 24 hours. Taken together, the accumulation of MSC-exo in the pathological brain was highly correlated with neuroinflammatory signals, suggesting that exosome migration was triggered by inflammation. All the results confirmed the application potential of exosomes as multifunctional theranostic agents in cerebrovascular and neurodegenerative diseases. Additionally, MSC-exo not only promoted neuronal survival and angiogenesis but also attenuated peripheral immunosuppression, which supported the use of MSC-exo in the treatment of stroke.

DCs are another promising source of exosomes for the treatment of neurological diseases. DC-derived exosomes (DC-Exo) are not only modulators of innate and adaptive immunity but also participate in antigen presentation. DC-Exo present CD86, CD80, major histocompatibility complex class I (MHC-I), and MHC-II on the membrane 128. As the composition of DC-Exo depends on external stimulation and cell state, primary DCs were cultured and stimulated with low levels of IFN-γ, and the subsequently released exosomes with high levels of miRNAs could reduce oxidative stress and increase CNS myelination *in vivo*. In addition, a decreased immune response in stroke patients may aggravate the prognosis 129. DC-Exo can be directly recognized by primed effector T cells or be captured and next presented by other DCs to activate naïve T cells, modulating the immune system during stroke 130. Neuroinflammation can be detected in various brain pathologies. Recent studies have demonstrated that neuroinflammation triggered naïve macrophage-derived exosomes (Mϕ-exo) to cross BBB and delivered the cargo protein (brain-derived neurotrophic factor, BDNF) to the inflamed brain. Innovatively, Haney MJ et al. developed a new Mϕ-exo-based delivery system with catalase loading to treat Parkinson's disease. In this study, polymeric PLGA NPs or liposomes with similar size to exosomes were also synthesized. After co-incubation with the target neuronal cell (PC12 cells), Mϕ-exo were effectively internalized into neurons and then built up on plasma membrane, showing an unparalleled accumulation compared to these synthetic particles 40. However, most intravenously administered exosomes show a predominant localization in the spleen followed by the liver 131. The insufficient targeting capability of unmodified exosomes still limits their clinical applications.

### Enhanced targeting and therapeutic effects of engineered exosomes

Through some simple, fast and effective methods, the targeted modification of exosomes can greatly improve the therapeutic effect. For example, Tian T et al. used c(RGDyK) peptide to recognize cerebral ischemia-induced expression of integrin α_v_β_3_ in cerebral vascular endothelial cells to achieve targeted therapy 21. It was calculated that 1 mg/mL MCS-exo contained 523 nM modified-peptide on average. In the mouse MCAO/R model, compared with unmodified exosomes, the engineered c(RGDyK)-conjugated exosomes (cRGD-Exo) were significantly concentrated in the ischemic brain lesion area. The fluorescence ratio of ipsilateral (lesion region) and contralateral (non-ischemic region) areas increased sharply to 11. The vessels in both penumbra and ischemic core showed a strong increase in integrin α_v_β_3_ expression. The study found that, cRGD-Exo loaded with Curcumin (cRGD-Exo-cur) significantly reduced expression levels of TNF-α, IL-1β and IL-6, activated microglia in the peri-infarct area, and induced their highly branched process. Compared with non-engineered exosomes (Exo-cur), cRGD-Exo administration could more effectively inhibit inflammation and apoptosis in the ischemic brain, meanwhile, there was no damage or function loss in liver and lung tissues. Kim HY et al. used MSC-derived magnetic exosomes with iron oxide nanoparticles (MVN) to improve the targeting and therapeutic effects in ischemic lesion 113. The ability of MNV to target endothelial cells and neurons might be attributed to the VLA-4 and SNAP-25 proteins expressed on the MSC membrane. Compared with natural exosomes, magnetic treated exosomes contained more therapeutic molecules (FGF2, Ang-1, VEGF, BDNF, HGF, and TGF-β3). After systemic injection of MVN in rats induced by transient MCAO, the magnetic navigation *via* neodymium magnets could increase the accumulation of exosomes in ischemic lesion by 5.1 times, and the fluorescence ratio of the left and right hemispheres increased to 9.2. The accumulated MNV in ischemic lesion induced the polarization of macrophages from the inflammatory M1 phenotype to the anti-inflammatory M2 phenotype in the brain. MNV treatment promoted angiogenesis, anti-inflammatory and anti-apoptosis, thereby significantly reducing infarct volume and improving motor function in the ischemic brain. Cui GH et al. bound the central system-specific targeting peptide RVG to the surface of MSC-Exo *via* DOPE-NHS linker 51. Through specific interaction with acetylcholine receptors, exosomes could effectively enter neuronal cells. In comparison with natural exosomes, the modification of RVG increased the distribution of MSC-Exo in the cerebral cortex and hippocampus by about 3-fold, and reduced the plaque deposition in hippocampus by 2-fold. MSC-RVG-Exo were superior to unmodified exosomes to significantly downregulate the levels of TNF-α, IL-β and IL-6, markedly increase the level of IL-10, and improve the cognitive function of AD mice. Jahangardd Y et al. transfected recombinant expression vectors carrying miR-29a or miR-29b precursor sequences into human embryonic kidney 293 cells (HEK-293T cells) and rat bone marrow mesenchymal stem cells (r-BMSCs) 132. Compared with that in the untransfected cells, the overexpression of miR-29a and miR-29b in exosomes from genetically engineered HEK-293T cells were increased by about 5.6-fold and 26.8-fold, respectively. The level of miR-29b in the purified exosomes from engineered r-BMSCs was increased by approximately 2.6-fold. *BACE1* is one of the target genes of miR-29s family and overexpressed in AD, and its down-regulation can reduce Aβ accumulation. Besides, miR-29s family is also a key survival factor for neuronal cells. In Alzheimer's disease rat model, bilateral injection of miR-29b-enriched exosomes into the hippocampal CA1 region (cornu ammonis area) resulted in the up-regulation of miR-29b and down-regulation of NAV3 and BIM. These engineered exosomes had protective effects against amyloid pathogenesis and could restore Aβ learning and memory deficits. Alvarez-Erviti L et al. achieved this result by engineering DCs to express RVG-Lamp2b, which could deliver GAPDH siRNA specifically to neurons, oligodendrocytes, and microglia in the brain 37. After systemic administration, exosome therapy could significantly knock down the mRNA and protein levels of *BACE1* in wild-type mice, up to 60%. The extremely low levels in the liver and other organs represented a major improvement over most current siRNA delivery strategies. Qu M et al. reported new dopamine CNS delivery formulations based on blood exosomes from reticulocytes 38. About 0.18 mg of blood exosomes (blood-exo) were purified from 10 mL of serum, containing about 7.49×10^10^ particles. When blood-exo were pre-incubated with transferrin for 4 h, the uptake of mouse brain microvascular endothelial cells (bEnd.3 cells) increased in a concentration-dependent manner. After intravenous injection, the concentration of dopamine encapsulated by blood-exo was 1.02±0.15 nmol/g in the brain, while it was not detectable after injection of free dopamine. The results showed that blood-exo administration could increase dopamine delivery to the brain by 15-fold and improve the disease phenotype in the PD model.

In short, the development of engineered exosomes with targeted functions for disease treatment can increase the accumulation of drugs in the location of brain disease lesions and reduce damage to the liver, kidney and other organs. This method overcomes the shortcoming of poor therapeutic effects caused by poor targeting of cerebral ischemic lesions after systemic administration. Therefore, the development of targeted parts for brain tissue will expand the application of exosomes in brain diseases. Their capacity to be appropriately designed, the lack of induction of the immune system have made exosomes an ideal carrier to deliver genetic materials and drugs to the central nervous system. Engineered exosomes not only retain their own functional molecules, but also benefit from their newly loaded molecules. The limitations of natural exosomes, including short half-life in circulation, low targeting efficiency, and low concentration of functional molecules can be eliminated after purposeful design.

### Translational possibility of engineered exosomes

As exosomes are highly complex vesicles involved in many pathological and physiological processes, their secretion is strictly regulated and influenced by external and internal stimuli including biotic and abiotic stresses. The composition of exosomes is not random, and each exosome contains specific molecular information. A clinical trial using allogenic mesenchymal stem cells derived exosomes enriched by miR-124 to promote neurovascular remodeling and functional recovery on disability of patients with acute ischemic stroke is in progress (https://clinicaltrials.gov/ct2/show/NCT03384433). Engineered exosomes have great potential in diagnosis, treatment, clinical transformation, but they still face some technical challenges. The following issues need be adequately addressed 1) scalability and production efficiency of exosomes, 2) standardization of exosomes isolation, quantification and characterization, 3) quality control of uniform exosomes, 4) storage conditions final exosome products, 5) database of absorption, distribution, metabolism and excretion of exosomes according to the cell source and administration route. At present, 3D extracellular matrix-based scaffolds have been developed to simulate the native environment of donor cells, and bioreactors have been used to for large-scale cell expansion, thereby achieving mass production of exosomes 133. A variety of isolation and characterization techniques are available to analyze exosomes, providing critical information about the purity of exosomes for future clinical treatment. In addition to conventional morphology, particle size, and protein analysis, it can also be accurate to RNA, lipid and metabolite analysis 134. Study on exosomes has gained increasing interest, and research data is rapidly accumulating. Researchers in this field are also evaluating and scoring the experimental parameters of exosomes in related articles in publications. The gradually establishing standardized database can help exosomes to shift from bench top science to translational and clinical area, and further be used for *in vivo* treatment and diagnosis 135, 136.

## Imaging techniques for exosome tracking

**Figure 5:**
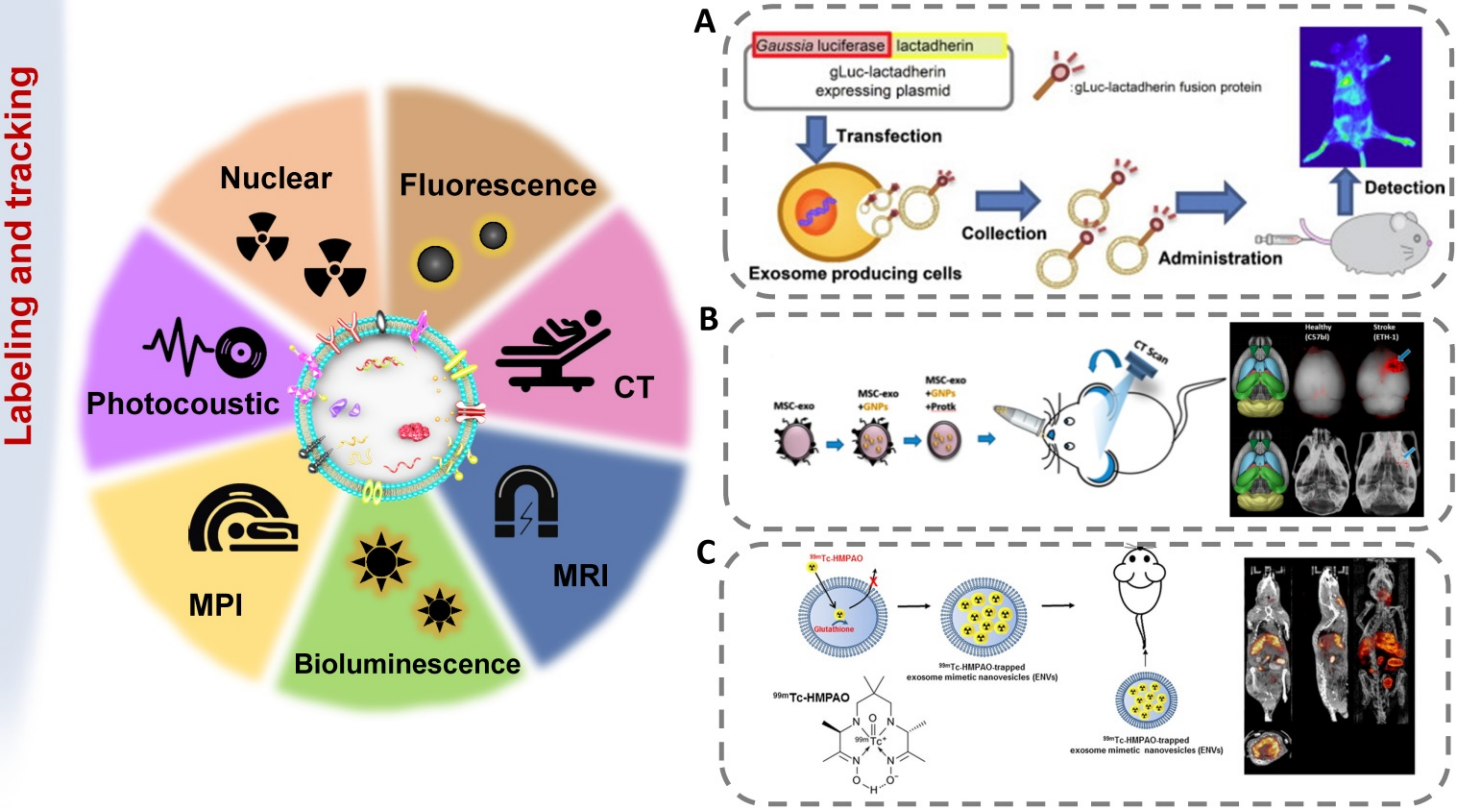
Imaging strategies for exosomes* in vivo* tracking. Various strategies have been developed to elucidate exosome trafficking and selective targeting *in vivo*. This will contribute to a better understanding of the biological role of exosomes and their potential as natural drug carriers. **(A)** Exosomes were successfully labeled with Gaussia luciferase-lactadherin fusion protein (adapted with permission from 140, copyright 2013 Elsevier). **(B)** *Ex vivo* fluorescent imaging and *in vivo* CT imaging of MSC-exo using PKH-26 and gold nanoparticles as labeling agents (adapted with permission from 141, copyright 2019 American Chemical Society). **(C)** ^99^mTc-hexamethylpropyleneamineoxime (HMPAO) was chosen as a proper radiotracer to monitor Exosome-mimetic nanovesicles (ENVs) (adapted with permission from 142, copyright 2015 Springer Nature).

The methods of labeling exosomes and tracking their biodistribution are very important for evaluating their therapeutic potential for brain diseases 137. There are still some challenges in tracking exosomes in organisms, including the small size of exosomes, the rapid dispersion in body fluids, the similar composition to human cells, and the lack of high-contrast imaging technology 138. The abovementioned genetic, chemical or biological modification of electric vehicles not only expands, changes or enhances their therapeutic capabilities but also brings convenience to imaging. 139. In order to track exosomes *in vivo*, various strategies have been developed to effectively label exosomes (Figure 5). The use of molecular imaging techniques, such as fluorescence imaging, bioluminescence imaging, radionuclide and magnetic resonance imaging, to noninvasively monitor the absorption, distribution, metabolism and excretion of exosomes can provide valuable data for CNS diseases treatment.

### Fluorescence labeling and tracking

Fluorescence imaging (FLI) is a simple, efficient and relatively low-cost imaging technique commonly used to track exosomes. It is excited by excitation light (visible light, near-infrared (NIR) light) by a specific probe and emitted below the excitation energy. Thus, the excitation light of a specific wavelength could be detected efficiently and noninvasively 143. In the spectral range of 650-950 nm, biological tissue has extremely low autofluorescence and high tissue penetration, which can achieve maximum light penetration, thereby achieving a high signal-to-noise ratio and high sensitivity 144. However, because free lipophilic dyes have long fluorescent signals, labeling lipids may result in inaccurate measurements of labeled exosome half-lives. The fluorescence resolution is low, which leads to the limitation of the depth of light penetration, so the imaging is limited to the superficial tissue in the millimeter range 145.

### Bioluminescence labeling and tracking

Another way to track exosomes is bioluminescence imaging (BLI) and light produced by natural biological processes (luciferase-substrate reactions). Bioluminescence is the emission of bioluminescence through the reaction of a substrate with ATP and Mg^2+^ or oxygen alone, without the need for an excitation source to emit light 146. Takahashi Y et al. first revealed that bioluminescent reporter protein-labeled exosomes could achieve *in vivo* visualization 140. Continuous *in vivo* imaging showed that B16-BL6 exosomes disappeared from the blood circulation quickly, with a half-life of approximately 2 min. In addition, B16-BL6 exosome-derived signals were first distributed to the liver and then to the lung. After that, Takahashi Y et al. labeled exosomes with a fusion protein of Gaussian luciferase and gLuc-lacherherin and evaluated the tissue distribution of exosomes in mice by bioluminescence detection 147. BLI technology provids a whole-body map of the exosome biodistribution. As the markers are inherent, exosome tracking by BLI is very reliable.

### Radionuclide and magnetic resonance labeling and tracking

Although fluorescent imaging is a convenient tool, radioisotope labeling can further detect exosomes in deep organs. The distribution of exosomes in tissues was quantitatively evaluated by measuring radioactivity with a gamma counter. Morishita M et al. solved the reduction in the gLuc-latex mucin luminescence signal *in vivo* by combining ^125^I-labeled biotin derivatives with the exosomes of B16BL6 cells transfected with streptavidin-latex mucin protein particles 148. After intravenous injection of ^125^I-labeled B16BL6 secreted exosomes into mice, the radioactivity quickly disappeared from the blood circulation. After 4 h, about 1.6%, 7%, and 28% of the injected radiation intensities were detected in the spleen, lung and liver, respectively. The liver was the main metabolic organ for eliminating exogenously administered B16BL6-derived exosomes. These results indicated that this labeling method could be used to quantitatively evaluate the amount of administered exosomes distributed to each organ. Varga Z et al. used the ^99m^Tc-tricarbonyl complex to label erythrocyte-derived exosomes, which were found to show stability in mice by SPECT/CT imaging 149. MRI has the advantages of no radiation and high spatial resolution. When exosomes are tracked with MRI, the exosomes must be labeled with a magnetic contrast agent, such as superparamagnetic iron oxide nanoparticles 150. Bose RJC et al. fused 70 nm gold iron nanoparticles (GIONs) with exosomes through a top-down process, successfully labeled the exosomes, and performed *T2*-weighted MR imaging on the 12^th^ day after systemic administration 151.

Fluorescence-based *in vivo* imaging techniques (FLI, BLI) are highly limited in resolution and penetration depth. They are convenient to be used in cell-level experiments* in vitro*, but *in vivo* results still rely on *ex vivo* imaging combined after subsequent anatomical separation, so they are more suitable for pre-clinical research. Other tracking methods with high resolution and maximum penetration of deep structures are apt to apply from research to clinical application. The summary of the tracking techniques of exosomes in cerebrovascular and neurodegenerative disease therapies is shown in Table 3.

## Conclusion and perspectives

Over the last decade, researchers have made great progress in understanding the biogenesis, molecular content and biological function of exosomes. Accumulating evidence suggests that the composition of exosomes is driven by the type and physiological state of the source cells. Exosomes secreted by healthy cells may have therapeutic potential comparable to the cells themselves, suggesting the importance of identifying proper cellular sources. In cerebrovascular and neurodegenerative disease models, exosomes present a pathologically specific distribution in the brain. The engineering technology of exosomes can effectively improve the targeting rate and reduce the required dose, which may be the key to their clinical application. This review aims to promote the successful transformation of exosomes into clinical applications. On the one hand, an appropriate administration route can be selected to achieve a more optimized therapeutic effect; on the other hand, labeling and tracing can be used to obtain an in-depth understanding of the distribution and fate of various exosomes *in vivo*.

Overall, engineered exosomes are promising, noncellular, and modifiable extracellular vesicles that can improve the prognosis of patients with cerebrovascular and neurodegenerative diseases. As a cell-free therapy, exosomes would also minimize safety concerns about injecting live cells. However, before the widespread use of exosomes in clinical practice, there are still several limitations and challenges that need to be overcome. First, there is still no definite optimal purification technique for isolation of exosomes with high purity. The existing methods for separation, quantification and characterization of exosomes are quite different, making it hard to contrast and compare the research progress in this field. Second, deep divisions remain in the dosage, measurement standards, and administration routes of exosomes. Third, exosomes may show adverse effects when used in combination with therapeutic cargos. The heterogeneous components within exosomes may also lead to immunogenicity effects (immunostimulation or immunosuppression) according to the nature of donor cells. Fourth, the source cells of exosomes have different physiological states, which may affect the composition and therapeutic effects of exosomes derived from them. Considering the above factors and concerns, the quality control (QC) standard of derived exosomes should be established. The *in vivo* trafficking, biological fate of exosomes and their effects on target organs also need to be fully understood.

Despite the existing obstacles, using exosomes as potential biomarkers and therapeutics is attractive in various fields, including cancer, musculoskeletal diseases, and heart failure. Especially, in refractory neurological diseases, the future clinical transformation of exosomes is inspiring.

## Figures and Tables

**Table 1:** Summary of exosome administration routes in brain diseases

Source cells	Cargo	Administration route & pros and cons	Disease model	References
MSCs	—	*i.v.* most widely used, easy to operate, distribute throughout the body; easily be cleared by macrophages, short half-life index.	Intracerebral hemorrhage	28
MSCs	miR-124		Ischemic stroke	29
MSCs	miRNA-17-92		Ischemic stroke	30
MSCs	miR-210		Ischemic stroke	31
MSCs	HMGB1-siRNA		Ischemic stroke	32
MSCs	enkephalin		Ischemic stroke	33
ASCs	miR-126		Ischemic stroke	34
Mϕ	—		Ischemic stroke	35
blood plasma	quercetin		Alzheimer's disease	36
DCs	GAPDH siRNA		Alzheimer's disease	37
fresh serum	dopamine		Parkinson's disease	38
MSCs	curcumin	*i.v., i.n.* bypass the BBB, non-invasive, high bioavailability; small dose, limited olfactory epithelial surface area, short drug absorption and retention time, impact on nasal secretions.	Ischemic stroke	21, 39
Mϕ	catalase		Parkinson's disease	40
EL-4 T cells	curcumin		Brain inflammation	41
MSCs	—	*i.c.* direct efficacy, shorten drug retention time, reduce drug dose; high operation requirements, image guidance, wound pain, risk of surgical complications.	Ischemic stroke	42
bEnd.3 cells	—		Ischemic stroke	43
Mϕ	curcumin		Alzheimer's disease	44
MSCs, HEK-293T cells	miR-29b		Alzheimer's disease	45
Mϕ	neurotropic factor		Parkinson's disease	46
HEK-293T cells	catalase mRNA		Parkinson's disease	47

MSCs: mesenchymal stem cells; Mϕ: macrophages; ASCs: Adipose stem cells; DCs: dendritic cells. *i.v.*: intravenous injection; *i.n.*: intranasal injection; *i.c.*: intracerebral injection.

**Table 2:** Summary of engineered exosomes for brain targeting

Targeting agent	Targeting point	Modification method	Exosome source cells	Administration route	Disease model	References
CXCR4	CXCL12 in ischemic brain tissue	genetic engineering	MSCs	*i.c.*	Ischemic stroke	115
RVG	nicotinic acetylcholine receptor on neuronal cells	biochemical engineering	MSCs	*i.v.*	Alzheimer's disease	51
RVG-Lamp2b	acetylcholine receptor of neurons, Neuro2A cells, neural infarct site, neuroinflammation site	genetic engineering	MSCs	*i.v.*	Ischemic stroke	29
		genetic engineering	HEK293T cells	*i.v.*	Ischemic stroke	32
		genetic engineering	HEK293T cells	*i.v.*	Morphine relapse	116
		genetic engineering	HEK293T cells	*i.c.*	Parkinson's disease	47
		genetic engineering	DCs	*i.v.*	Alzheimer's disease	37
GNSTM-RVG-Lamp2b	acetylcholine receptor, bEnd.3 cells	genetic engineering	HEK293T cells	*i.v.*	—	117
c(RGDyK)	α_v_β_3_ integrin, microglia, neurons and astrocytes	biochemical engineering	MSCs	*i.v.*	Ischemic stroke	21, 31
		physical engineering	K562 CML cells	*i.v.*	—	118
IONP	magnetic field position	physical engineering	MSCs	*i.v.*	Ischemic Stroke	113
Tf	transferrin receptor	genetic engineering	MSCs	*i.v.*	Ischemic Stroke	33
		physical engineering	blood	*i.v.*	Parkinson's disease	38

RVG: rabies virus glycoprotein; GNSTM: glycosylation-stabilized peptide; RVG-Lamp2b: RVG fused to Lamp2b; GNSTM-RVG-Lamp2b: GNSTM and RVG fused to Lamp2b; CXCR4: CXC motif chemokine receptor type 4; IONP: iron oxide nanoparticles; Tf: transferrin. MSCs: mesenchymal stem cells; Mϕ: macrophages; ASCs: Adipose stem cells; DCs: dendritic cells. *i.v.*: intravenous injection; *i.n.*: intranasal injection; *i.c.*: intracerebral injection.

**Table 3:** Summary of exosomes labeling and *in vivo* imaging techniques

Imaging technique	Advantages	Disadvantages	Labeling agent	Exosome source cells	Administration route	Disease model	References
FLI	low cost, multiple colors, high-throughput efficiency	low sensitivity, low quantitative capacity, poor tissue penetration depth	Cy5.5, DiI	MSCs, NSCs, Mϕ	*i.v.*	Ischemic stroke	21, 29, 31, 35, 152
			PKH26, curcumin	MSCs, Mϕ	*i.v., i.p.*	Alzheimer's disease	44, 153
			DiD, DiI,	fresh serum, Mϕ	*i.v., i.n.*	Parkinson's disease	38, 40
BLI	low background signal, high sensitivity, independence from excitation source	need of substrate injection before each imaging, short half-life of the substrate	Gluc	F11 cells	—	Neurogenesis	154
CT	deep brain tissue imaging to avoid skull shielding of light signals	ionizing radiation	GNP	MSCs	*i.v., i.n.*	Ischemic stroke, Parkinson's disease, Alzheimer's disease, autism disorder	72, 155
MRI	high spatial resolution and excellent soft tissue contrast into deep structures, rapid *in vivo* acquisition, no radiation burden	difficulties to distinguish clear location and quantitatively understand *in vivo* fate	FTH1, USPIO	MSCs, ASCs	*i.m.*	—	156, 157
RNI	quantitative capability, excellent sensitivity and good penetration into deep tissues	inferior spatial resolution, toxicity caused by repeated radiation exposure	^125^Iodine	B16BL6 cells	*i.v.*	—	24, 142
			^99m^Tc-HMPAO	Mϕ	*i.v.*	—	142

FLI: fluorescence imaging; BLI: bioluminescent imaging; CT: computed tomography; MRI: magnetic resonance imaging; RNI: radionuclide imaging. GNP: gold nanoparticles; USPIO: ultra-small superparamagnetic iron oxide nanoparticles; FTH1: ferritin heavy chain; MSCs: mesenchymal stem cells; NSCs: neural stem cells; Mϕ: macrophages; ASCs: Adipose stem cells; DCs: dendritic cells. *i.v.*: intravenous injection; *i.p.*: intraperitoneal injection;* i.n.*: intranasal injection; *i.c.*: intracerebral injection; *i.m.*: intramuscular injection.
